# Airway reconstruction: review of an approach to the advanced-stage laryngotracheal stenosis^☆^

**DOI:** 10.1016/j.bjorl.2016.03.012

**Published:** 2016-04-27

**Authors:** Mohamad Ahmad Bitar, Randa Al Barazi, Rana Barakeh

**Affiliations:** a American University of Beirut, Faculty of Medicine and Medical Center, Department of Otolaryngology and Head & Neck Surgery, Beirut, Lebanon; b American University of Beirut, Faculty of Medicine and Medical Center, Department of Pediatrics and Adolescent Medicine, Beirut, Lebanon; c University of Sydney, Sydney Medical School, The Children's Hospital at Westmead, Department of ENT Surgery, Sydney, Australia; d Al Jalila Children's Specialty Hospital, Department of Otolaryngology Head & Neck Surgery, Dubai, UAE

**Keywords:** Laryngotracheal stenosis, Subglottic stenosis, Laryngotracheal reconstruction, Cricotracheal resection, Staging, Mapping, Estenose laringotraqueal, Estenose subglótica, Reconstrução laringotraqueal, Ressecção cricotraqueal, Estadiamento, Mapeamento

## Abstract

**Introduction:**

The management of laryngotracheal stenosis is complex and is influenced by multiple factors that can affect the ultimate outcome. Advanced lesions represent a special challenge to the treating surgeon to find the best remedying technique.

**Objective:**

To review the efficacy of our surgical reconstructive approach in managing advanced-stage laryngotracheal stenosis treated at a tertiary medical center.

**Methods:**

A retrospective review of all patients that underwent open laryngotracheal repair/reconstruction by the senior author between 2002 and 2014. Patients with mild/moderate stenosis (e.g. stage 1 or 2), or those who had an open reconstructive procedure prior to referral, were excluded. Patients who had only endoscopic treatment (e.g. laser, balloon dilatation) and were not subjected to an open reconstructive procedure at our institution, were not included in this study. Variables studied included patient demographics, clinical presentation, etiology of the laryngotracheal pathology, the location of stenosis, the stage of stenosis, the type of corrective or reconstructive procedure performed with the type of graft used (where applicable), the type and duration of stent used, the post-reconstruction complications, and the duration of follow-up. Outcome measures included decannulation rate, total number of reconstructive surgeries needed to achieve decannulation, and the number of post-operative endoscopies needed to reach a safe patent airway.

**Results:**

Twenty five patients were included, aged 0.5 months to 45 years (mean 13.5 years, median 15 years) with 16 males and 9 females. Seventeen patients (68%) were younger than 18 years. Most patients presented with stridor, failure of decannulation, or respiratory distress. Majority had acquired etiology for their stenosis with only 24% having a congenital pathology. Thirty-two reconstructive procedures were performed resulting in decannulating 24 patients (96%), with 15/17 (88%) pediatric patients and 5/8 (62.5%) adult patients requiring only a single reconstructive procedure. Cartilage grafts were mostly used in children (84% vs. 38%) and stents were mostly silicone made, followed by endotracheal tubes. The number of endoscopies required ranged from 1 to 7 (mean 3). More co-morbidities existed in young children, resulting in failure to decannulate one patient. Adult patients had more complex pathologies requiring multiple procedures to achieve decannulation, with grafting less efficacious than in younger patients. The pediatric patients had double the incidence of granulation tissue compared to adults. The decannulated patients remained asymptomatic at a mean follow-up of 50.5 months.

**Conclusion:**

The review of our approach to open airway repair/reconstruction showed its efficacy in advanced-stage laryngotracheal stenosis. Good knowledge of a variety of reconstructive techniques is important to achieve good results in a variety of age groups.

## Resumo

**Introdução:**

A conduta da estenose laringotraqueal é complexo e é influenciado por vários fatores que podem afetar o resultado final. Lesões em estágio avançado representam um desafio especial para o cirurgião encontrar a melhor técnica de tratamento.

**Objetivo:**

Avaliar a eficácia de nossa abordagem de reconstrução cirúrgica no tratamento de estenose laringotraqueal em estágio avançado em um centro médico terciário.

**Método:**

Revisão retrospectiva de todos os pacientes que foram submetidos a tratamento cirúrgico/reconstrução laringotraqueal aberta pelo autor principal, entre 2002 e 2014. Os pacientes com estenose leve (por exemplo, estágio 1 ou 2), ou aqueles submetidos a procedimento de reconstrução aberta antes da indicação, foram excluídos. Pacientes que tinham sido submetidos somente a tratamento endoscópico (por exemplo, laser, dilatação por balão) e não haviam sido submetidos a procedimento de reconstrução aberta em nossa instituição, não foram incluídos neste estudo. As variáveis estudadas incluíram dados demográficos dos pacientes, apresentação clínica, etiologia da doença laringotraqueal, local da estenose, estágio da estenose, o tipo de procedimento corretivo ou reconstrutor realizado com o tipo de enxerto utilizado (onde aplicável), tipo e duração do stent utilizado, complicações pós-reconstrução, e duração do seguimento. Os resultados incluíram taxas de decanulação, número total de cirurgias reconstrutoras necessárias para possibilitar a decanulação, e o número de endoscopias pós-operatórias necessárias para obter uma via aérea patente e segura.

**Resultados:**

Vinte e cinco pacientes foram incluídos, com idade de 0,5 meses a 45 anos (média de 13,5 anos, mediana de 15 anos) com 16 homens e 9 mulheres. Dezessete pacientes (68%) eram menores de 18 anos. A maioria dos pacientes apresentava estridor, falha de decanulação ou desconforto respiratório. A maioria das estenoses era adquirida, enquanto apenas 24% presentavam apresentavam causa congênita. Trinta e dois procedimentos reconstrutores foram realizados, resultando em decanulação de 24 pacientes (96%), com 15/17 (88%) pacientes pediátricos e 5/8 pacientes (62,5%) adultos que necessitaram de apenas um único procedimento reconstrutor. Enxertos de cartilagem foram utilizados principalmente em crianças (84% vs. 38%) e a maioria dos stents era feita principalmente de silicone, seguidos por tubo endotraqueal. O número de endoscopias necessárias variou de 1 a 7 (média de 3). Mais comorbidades foram observadas em crianças pequenas, resultando em falha de decanulação em um paciente. Pacientes adultos apresentavam doenças mais complexas que requereram vários procedimentos para decanulação, com enxertos menos eficazes do que em pacientes mais jovens. Os pacientes pediátricos apresentaram o dobro da incidência de tecido de granulação em comparação aos adultos. Os pacientes decanulados permaneceram assintomáticos em um seguimento médio de 50,5 meses.

**Conclusão:**

A revisão da nossa abordagem para tratamento cirúrgico/reconstrução aberta das vias aéreas demonstraram eficácia na estenose laringotraqueal em estágio avançado. O conhecimento de uma variedade de técnicas de reconstrução é importante para conseguir bons resultados em vários grupos etários.

## Introduction

A significant increase in the incidence of laryngotracheal stenosis (LTS) occurred after the advent of neonatal intubation in the 1960s as described first by McDonald and Stocks.^1^ However, over the past few decades, the incidence has decreased given the effort put in the education of the nursing and medical staff involved in endotracheal tube care and the development of new tube material.^2^ Laryngotracheal stenosis can be congenital or acquired and can affect the supraglottis, glottis, subglottis, the trachea, or a combination of these levels at the same time, although the most common location in children is the subglottis.^2^, ^3^

On the other hand, LTS in the adult population has a different spectrum of pathologies. The main cause of airway stenosis in adults has been reported by Pena et al., to be endotracheal intubation followed by laryngeal trauma, hamartoma and amyloidosis.^4^ As such, the trachea is the most common site to be affected (2ry to trauma from the tube's cuff) followed by the larynx.

The management of LTS can be challenging with multiple factors involved that can affect the ultimate prognosis. Treatment should be personalized as per the patient's characteristics. The most commonly used approach so far is laryngotracheal airway reconstruction (LTR). Other methods include laser ablation, and endoscopic balloon dilatation. The latter is usually used in patients with mild stenosis (stage 1 or 2), in early immature lesions or soon after an airway reconstruction procedure to prevent restenosis. Balloon dilatation has recently become popular and sometimes overused. We believe LTR is still the treatment modality of choice for mature and advanced LTS.

Our approach to LTS has been to adapt to each patient's type of pathology by relying on mapping the lesion preoperatively to choose the most appropriate corrective surgical technique for that particular patient. It also relies on using a staging system specific to each type of pathology to ensure proper documentation, transmission of information, and reporting of data. In this study we revisit the surgical treatment of LTS, by reviewing our experience and assessing the efficacy of our approach in managing advanced-stage laryngotracheal stenosis treated at a tertiary medical center.

## Methods

We performed a retrospective review of all patients who were managed by the senior author (MAB) for LTS between 2002 and 2014. The institutional review board approved the study (Ethical committee approval number OTO.MB.11). Patients with mild stenosis (e.g. stage 1 or 2), or those who had an open reconstructive procedure prior to referral, were excluded. Patients, who had only endoscopic treatment (e.g. laser, balloon dilatation) and were not subjected to an open reconstructive procedure at our institution, were not included in this study. Variables studied included patient demographics, clinical presentation, etiology of the laryngotracheal pathology, the location of the stenosis, the stage of the stenosis using various grading systems appropriate to the topography of lesion, the type of corrective or reconstructive procedure performed with the type of graft used (where applicable), the type and duration of stent used, the post-reconstruction complications, and the duration of follow-up.

Our adopted approach includes mapping the various encountered airway pathologies preoperatively. On presentation, all patients had a flexible fiberoptic nasopharyngolaryngoscopy performed to evaluate the patency of the upper airways and assess the mobility of the vocal cords. If the patient had already a tracheostomy in place, a flexible fiberoptic tracheoscopy was performed through the tracheostomy tube to assess the lower airways.

If not already available from the referring physician, a CT scan of the neck/chest was then ordered to study the extent of the lesion prior to further evaluation in the operating theater. Direct laryngoscopy and bronchoscopy was then undertaken for a final and direct mapping of the lesion. If possible distal endoscopy through the stenotic area was performed to mark the distal part of the stenosis. The location of the tracheostomy tube (if present) with respect to the stenotic segment was also assessed. The final topography of the lesion was delineated by combining the results of all the above and recorded in the chart; including location, thickness, and length.

To better document the findings and properly transmit the information to other physicians, we classified the studied stenoses using lesion-appropriate staging systems. These included; the Cotton–Myer staging system^5^ for isolated Subglottic Stenosis (SGS), the Cohen's classification^6^ for Anterior Glottic webs/stenosis (AGS), the Bogdasarian–Olson classification^7^ for Posterior Glottic webs/stenosis (PGS) and the McCaffrey staging system^8^ for LTS.

The Cotton–Myer staging system^5^ describes the stenosis based on the percent relative reduction in cross-sectional area of the subglottis and it consists of four grades:

Grade I – less than 50% obstruction;
Grade II – 51–70% obstruction;
Grade III – 71–99% obstruction;
Grade IV – no detectable lumen or complete obstruction.

Cohen proposed the classification for anterior glottic web/stenosis^6^:

Type I – involvement of 35% or less of the glottis with little or no subglottic involvement;
Type II – involvement of 35–50% of the glottis with minimal subglottic involvement;
Type III – involvement of 50–75% of the glottis extending to the lower border of the cricoids;
Type IV – a thick web covering 75–90% of the glottis and extending to the lower border of the cricoids.

On the other hand, Bogdasarian and Olson classified the extent of posterior glottic web/stenosis into four types^7^:

Type I – vocal process adhesion;
Type II – posterior commissure stenosis with scarring in the inter-arytenoid plane and internal surface of the posterior cricoid lamina;
Type III – posterior commissure stenosis with unilateral cricoarytenoid joint ankylosis;
Type IV – posterior commissure stenosis with bilateral cricoarytenoid joint ankylosis.

The McCaffrey staging system^8^ was developed and used for LTS in the adult patient. Though it was not validated to measure the outcome in the pediatric age group, we opted to use it just for documentation because of lack of a similar system in children. The McCaffrey staging system is divided into four stages describing the site of stenosis:

Stage I – lesions confined to the subglottis or trachea that are less than 1 cm long;
Stage II – subglottic lesions longer than 1 cm within the cricoid ring and not extending to the glottis or trachea;
Stage III – subglottic lesions extending into the upper trachea but not involving the glottis;
Stage IV – lesions involving the glottis with fixation or paralysis of one or both vocal cords.

The classification of an isolated tracheal pathology was hard as there is no specifically adopted staging system for that location. We have adopted the classification used by Anand et al.^9^ to stratify the managed tracheal pathologies. The lesion is classified depending on its location (cervical vs. thoracic), length (1–3 cm vs. > 3 cm) and severity of obstruction (mild, moderate or severe).

The outcome was measured by the decannulation rate, the total number of reconstructive procedures required to achieve decannulation, and the number of post-reconstructive endoscopies needed to reach a safe patent airway.

The type of surgery performed on each patient was tailored according to the preoperative mapping of the lesion, and the stability of the laryngotracheal framework.

Supraglottic stenosis’ correction (what we like to call “supraglottic reconstruction”) was the most challenging procedure which always involves stenting and requires close follow-up.

Laryngotracheal reconstruction was used to expand the glottic, subglottic or laryngotracheal stenotic segment. The framework should be stable enough to accommodate an inserted graft. The expansion may be anterior, posterior or both depending on the topography of the stenosis at a particular site. The tracheal segment of a combined stenosis (i.e. laryngotracheal) can be shortened by excising it if needed to limit the number of used grafts or if it is circumferential as the graft will only expand the anterior part of the tracheal segment.

Cricotracheal resection (CTR) was used in advanced stage LTS, in cases where the framework was unstable due to replacement of the cartilage with fibrosis, in adults where the ossification of the rib cartilage and the airway framework was present, and in revision LTR's where grafts were previously tried.

Tracheal resection and anastomosis was used to remove an isolated segment of the trachea where circumferential stenosis exists.

The reconstructive procedure was sometimes a single stage where the patient did not need a tracheostomy present postoperatively. This was feasible in cases where the stenotic lesion was excised or expanded and the resultant reconstruction was stable enough to require short or no stenting in the postoperative period. At other times, a double stage was needed, where decannulation was performed after ensuring that the reconstructed area healed properly and the airway was safe.

As the studied population included both pediatric and adult patients, we further analyzed the results as two separate series to compare patients’ characteristics, preoperative findings, the type of surgeries needed and their outcome.

## Results

We reviewed 25 patients aged 0.5 months to 45 years (mean 13.5 years, median 15 years) with 16 males and 9 females. Seventeen patients (68%) were younger than 18 years. The clinical presentation was variable among the studied patients; 36% presented with stridor, 28% were referred for failure of decannulation, 20% presented with history of respiratory distress, 8% were seen for failure of intubation, and 8% complained of inability to swim. Most patients had an acquired cause with only 24% having a congenital pathology (Table 1).

**Table 1:** Etiology of the airway pathology.

Etiology	Number of patients
*Acquired*	19

*Prolonged intubation*	13
Polytrauma	7
Neurological disorder	3
Respiratory failure^a^	1
Suicidal attempt	1
Post-operative complication^b^	1

*Non-closure of tracheostomy site*	3
*Tracheal tear*^c^	1
*Chemical injury*	2
*Congenital*^d^	6
Total	25

a Patient had congenital heart disease.

b Patient had subglottic stenosis following intubation for rhinoplasty at another institution.

c Secondary to traumatic bronchoscopy while removing an aspirated foreign body at another institution.

d All were pediatric patients.

**Figure 1:**
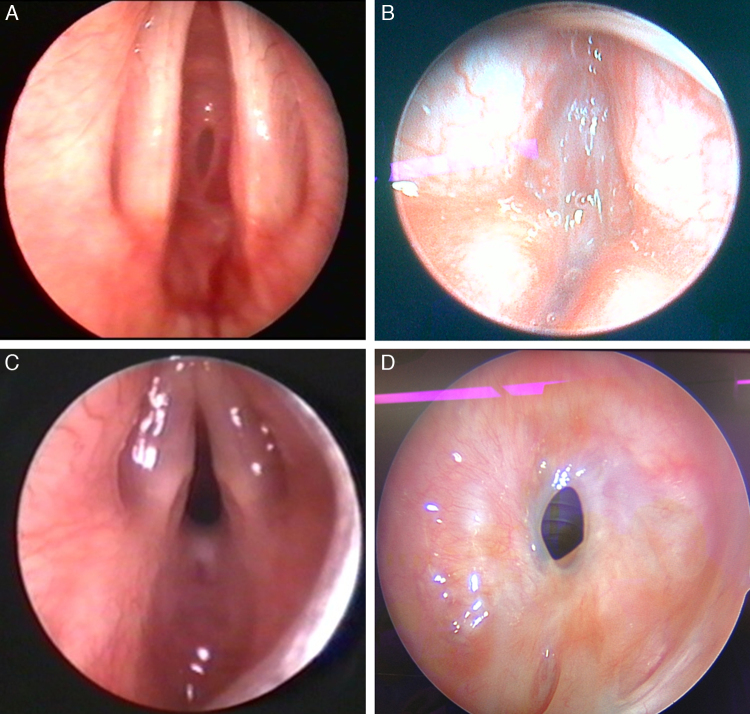
Mapping of various pathologies. (A) Grade 3 isolated subglottic stenosis; (B) type 4 glottic web; (C) type 4 posterior glottic stenosis; (D) cervical, moderate, 1–3 cm isolated tracheal stenosis.
Anterior glottic/subglottic (12%) – 2 patients had Cohen's type 4 lesion while one had type 3 (Fig. 1B);
Posterior glottic/subglottic (12%) – all had a type 4 Bogdassarian & Oslon lesion (Fig. 1C);
Tracheal (16%) – 5 patients had isolated tracheal lesions; four had a cervical tracheal pathology while one had a thoracic location. One had severe, two moderate and two mild narrowing. Three of the patients had a lesion affecting >3 cm of the tracheal length while the other 2 had a lesion involving 1–3 cm of the trachea (Fig. 1D);
Laryngotracheal (20%) – 4 patients with LTS had McCaffrey stage 3, while one had stage 4;
Supraglottic (4%).

After preoperative mapping, we could classify the lesions as:

Subglottic (36%) – all had Myer–Cotton grade III (Fig. 1A);

Thirty-two open reconstructive procedures were performed on 25 patients (Table 2, Table 3). Sixteen patients had already had a procedure attempted prior to an open surgical reconstruction whether it was a tracheotomy, balloon dilatation, or laser treatment. All the intraoperative findings corresponded to the topography resultant from the preoperative mapping.

**Table 2:** Reviewed pediatric patients with advanced laryngeal and or tracheal stenosis.

*N*	Age	Co-morbidities	Lesion	Stage	Tracheostomy timing	Procedures	Stenting (type/duration/g.t.)	Number of endoscopies needed after each surgery	Outcome
1	12d	Cardiac anomalies	SGS	Cotton Myer III	None	LTR + AG (SS)	ET Tube-5 days-No	Three	Decannulated
2	3m	Sturge-Weber syndrome	SGS	Cotton Myer III	During the 1st procedure	Endoscopic CO2 laser ablation			Decannulated
		Subglottic hemangioma				LTR + AG (SS)	ET tube-5 days-No	Two	
3	8m	Congenital TOF	SGS	Cotton Myer III	Prior to the procedure	LTR + APG (DS)	Silicone^a^ 5 days–Yes	Seven	Decannulated
		Esophageal atresia							
		Duodenal atresia							
4	1y	None	AGS	Cohen IV	Prior to the 1st procedure	Anterior cricoid split + AG (DS)	Keel-19 days-Yes-Mitomycin	Four	Decannulated
						LTR + AG (DS)	Silicone-12 days-Yes-Mitomycin	Five	
5	2y	Seizures	SGS	Cotton Myer III	Prior to 1st the procedure	LTR + APG (DS)	Silicone-7 days-No	Four	Decannulated
						LTR + AG (SS)	ET tube-3 days-No	Two	
6	3y	Bilateral severe hearing loss	PGS	Bogdassarian Olson IV	During the procedure	LTR + APG (DS)	Silicone-21 days-Yes	Four	Decannulated
7	5y	None	Tracheal	Anand (tracheal, moderate, >3 cm)	After the procedure^b^	Primary repair through Thoracotomy (DS)	ET tube-11 days-No	Three	Decannulated
8	6y	Cerebral palsy	LTS	Mc-Caffrey III	Prior to the procedure	LTR + AG (DS)	None	One	Not decannulated
9	9y	None	SGS	Cotton Myer III	During the 1st procedure	Endoscopic dilatation			Decannulated
						LTR + APG (DS)	Silicone-7 days-Yes	Two	
10	9y	None	LTS	Mc-Caffrey III	Prior to the 1st procedure	Endoscopic dilatation			Decannulated
						LTR + AG (DS)	Abulkheir-5 days-No	Five	
						Endoscopic dilatation			
11	12y	None	Tracheal	Anand (cervical, severe, >3 cm)	Prior to the procedure	R + A (SS)	ET Tube-8 days-No	Two	Decannulated
12	13y	None	PGS	Bogdassarian Olson IV	None	LTR + PG (SS)	ET tube-7 days-No	One	Decannulated
13	15y	None	SGS	Cotton Myer III	Prior to the procedure	LTR + APG (DS)	Silicone-21 days-Yes	Two	Decannulated
14	15y	Down syndrome	SGS	Cotton Myer III	Prior to the procedure	LTR + AG (DS)	None-Yes	Five	Decannulated
15	15y	Mild mental retardation post trauma (car accident)	PGS	Bogdassarian Olson IV	During the procedure	LTR + APG (DS)	Silicone-25 days-Yes	Three	Decannulated
16	16y	None	Tracheal	Anand (cervical, mild, 1–3 cm)	None	Tracheoplasty + AG (SS)	None	One	Decannulated
17	17y	None	AGS	Cohen III	During the 2nd procedure	Endoscopic excision of web			Decannulated
						LTR + AG (DS)	Keel-27 days-Yes-Mitomycin	Three	
						Endoscopic excision of web			

SGS, subglottic stenosis; LTR, laryngotracheal reconstruction; AG, anterior graft; APG, anterior and posterior grafts; CTR, cricotracheal resection; R + A, resection and anastomosis; PG, posterior graft; ET, endotracheal tube; SS, single stage; DS, double stage; g.t., granulation tissue.

a Silicone stent is made of one of the flanges of a Montgomery T-tube, it is always plugged caudally to avoid aspiration with the upper tip placed just above the level of the vocal cords.

b Tracheotomy was performed after ET tube removal to help toileting and avoid prolonged intubation.

**Table 3:** Reviewed adult patients with advanced laryngeal and or tracheal stenosis.

*N*	Age	Co-morbidities	Lesion	Stage	Tracheostomy timing	Procedures	Stenting (type/duration/g.t.)	Number of endoscopies needed after each surgery	Outcome
1	18y	None	LTS	Mc-Caffrey III	During the 3rd procedure	R + A (SS)	ET Tube 1 day-No	One	Decannulated
						Endoscopic Dilatation			
						LTR + APG (DS)	Montgomery T-tube 21 days-No	Five	
						Tracheoplasty + AG (DS)	Silicone^a^ 40 days-Yes	One	
2	18y	Vocal cords paralysis	LTS	Mc-Caffrey IV	Prior to the 1st procedure	CTR (DS)	None	Three	Decannulated
						Right Posterior cordotomy			
3	18y	None	Supra-glottic	NA	Prior to the 1st procedure	Supraglottic reconstruction (DS)	Silicone 21 days-No	One	Decannulated
						Release of adhesions			
						Release of adhesions			
4	22y	None	Tracheal	Anand (cervical, mild, 1–3 cm)	None	Tracheoplasty + AG (SS)	ET tube 1 day-No	One	Decannulated
5	23y	None	LTS	Mc-Caffrey III	Prior to the 1st procedure	R + A (DS)	Montgomery T-tube 7 days-No	Two	Decannulated
						LTR + APG (DS)	Silicone-17 days-No	Three	
6	25y	None	SGS	Cotton Myer III	Prior to the procedure	LTR + APG (SS)	ET Tube 4 days-No	Three	Decannulated
7	29y	GERD	AGS	Cohen IV	Prior to the procedure	CTR + AG (DS)	None-Yes	Three	Decannulated
8	45y	None	SGS	Cotton Myer III	During the 3rd procedure	Endoscopic Dilatation			Decannulated
						LTR + APG (SS)	ET tube 5 days-No	Four	
						LTR + APG (DS)	Silicone 19 days-No	Two	
						Endoscopic dilatation			
						CTR (DS)	None-Yes-Mitomycin	One	

SGS, subglottic stenosis; LTR, laryngotracheal reconstruction; AG, anterior graft; APG, anterior and posterior grafts; CTR, cricotracheal resection; R + A, resection and anastomosis; PG, posterior graft; ET, endotracheal tube; SS, single stage; DS, double stage; g.t., granulation tissue.

a Silicone stent is made of one of the flanges of a Montgomery T-tube, it is always plugged caudally to avoid aspiration with the upper tip placed just above the level of the vocal cords.

**Figure 2:**
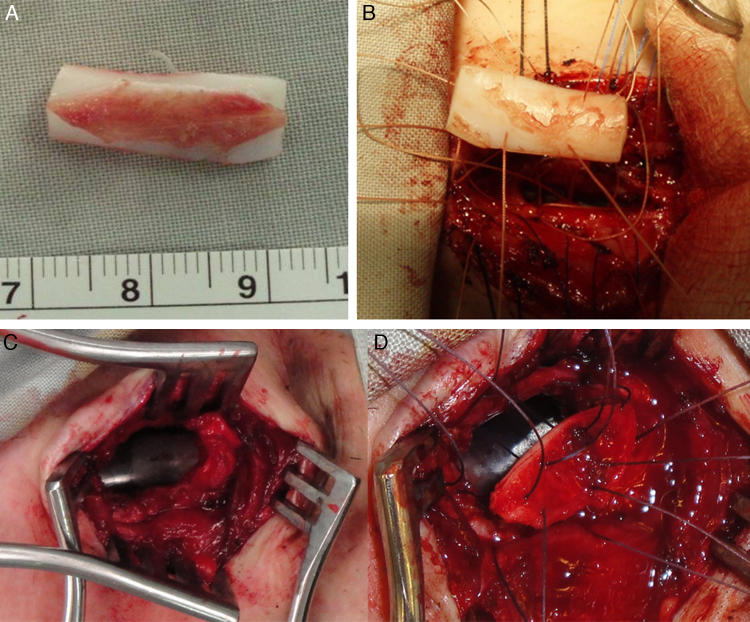
Grafting. (A) LTR using a modified boat for anterior grafting in a 15yo Down syndrome girl with anterior SGS 2ry to a high long-standing high tracheostomy. (B) Graft being fixed to the expanded cricoid cartilage. (C) Anterior tracheal defect 2ry to loss of cartilage and formation of fibrosis (that was excised) 2ry to a traumatic and long-standing tracheostomy in a 16yo boy. (D) Reconstruction using auricular cartilage graft.

**Figure 3:**
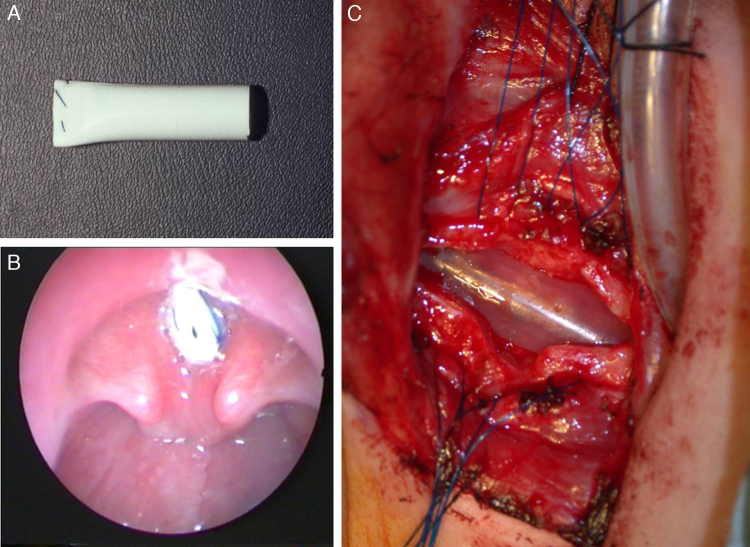
Stenting. (A) Silicone stent superiorly sutured to avoid aspiration during feeding. (B) Stent in place with upper end placed above the vocal cords to avoid inducing subglottic granulation tissue in a 3yo boy with posterior glottic stenosis. (C) Intraoperative view of an inserted stent in a 1yo with congential SGS.

Cartilage grafts were used to expand the airway when needed; these were mainly cartilage rib grafts (for cricoid expansion), conchal graft (for tracheal expansion) and thyroid alar graft (in infants) (Fig. 2). Stenting was needed to support the reconstructed area in 84% of the performed procedures. The stents were different in types and included silicone stents (part of Montgomery T-tube), endotracheal tubes, Aboulker stents, Montgomery T-tubes, and keels (Fig. 3). The duration of stenting varied from one to 40 days with a mean of 12.5 and a median of 14.5 days.

The outcome of the various corrective procedures was assessed based on the decannulation rate, and the number of needed corrective procedures to achieve decannulation. The number of needed endoscopy was also calculated and was not found to correlate with the degree of stenosis or type of surgery performed. Twenty four out of 25 patients were eventually decannulated (96%). Most patients required only one reconstructive procedure (80%) to achieve that. The number of endoscopies required to follow-up on the reconstructive procedures ranged from 1 to 7 with a mean of 2.8 and a median of 3. A general comparison between pediatric and adult patients is summarized in Table 4.

**Table 4:** Comparison between pediatric and adult groups.

	Pediatrics	Adults
No. of patients	17	8
Mean age (y)	8.2	25
Comorbidity	8 (47%)	2 (25%)
Glottic/SGS	12 (71%)	3 (38%)
No of procedures	19	13
2 procedures needed	2	1
3 procedures needed	0	2
Grafting used	16 (84%)	5 (38%)
Mean No of follow-up endoscopy	3	3
Incidence of granulation tissue	9 (47%)	3 (23%)
Mean duration of stenting (days)	12	14
Frequency of stent's using	16 (84%)	10 (77%)
Stent made of silicone	9	6

The most common complication was granulation tissue formation, which affected mainly patients with stents (75%). Postoperative complications are summarized in Table 5, along with the interventional steps taken to remedy them and their effect on the decannulation rate of the patients.

**Table 5:** Complications encountered postoperatively and their effect on decannulation.

Type of complication	No. of patients		Intervention		Eventually decannulated
	Pediatric	Adult	Type	No. of patients	
Granulation tissue formation	9	3	Inhaled steroids	2	2/2
			Excision	11	11/11
			Mitomycin C	4	4/4
Tracheomalacia^a^	4	0	No intervention	4	4/4
Infection and extrusion of graft^b^	0	1	Removal of graft	1	1/1
Re-stenosis	1	3	Surgical correction	4	4/4
Persistence of hoarseness^c^	1	0	No intervention	1	1/1
T-tube obstruction	0	2	Tube cleaning	1	1/1
			Tube removal	1	1/1

a Mild in nature (patients 2, 3, 5, 14 in Table 2).

b Posterior graft in patient 8 – Table 3.

c Patient had severe glottic/subglottic stenosis with significant involvement of the vocal cords (patient 4 – Table 2).

The decannulated patients remained asymptomatic at a mean follow-up of 50.5 months. They had good exercise tolerance and were able to carry on their normal daily activities (where applicable). No objective testing (e.g. pulmonary function test) was performed on these patients as they had no clinical indication for it.

The voice was evaluated postoperatively in our patients by the speech pathologist. The assessment evaluated the need for speech therapy or other measures in case the voice was not adequate and or not acceptable to the patient and or corresponding parents. All patients with SGS or posterior glottic/subglottic stenosis had a normal voice, even those patients who needed more than one procedure.

**Figure 4:**
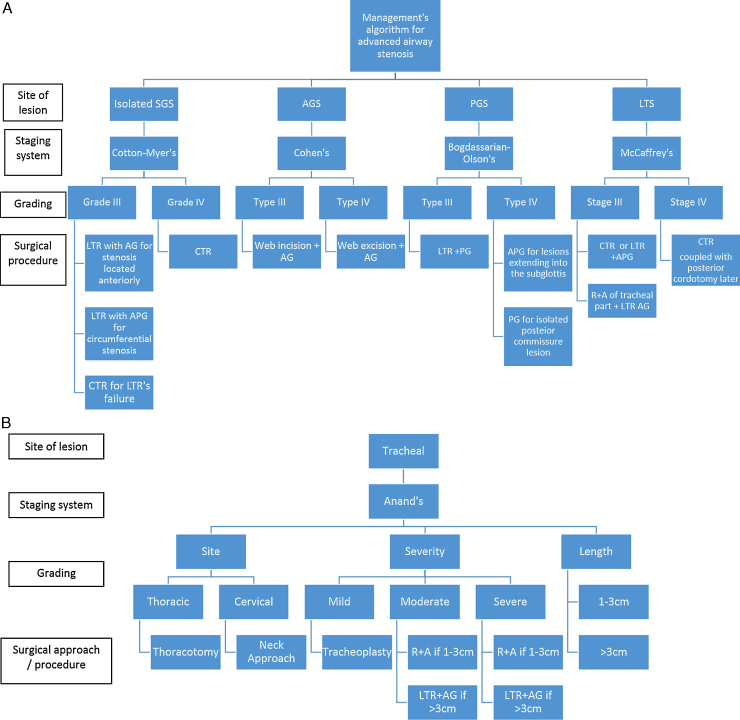
(A) and (B) Suggested algorithm to follow when managing advanced airway stenotic lesions. The key is to map the lesion first, stage it properly and then tailor the surgical procedure accordingly.

We have devised an algorithm to manage advanced laryngeal and or tracheal stenosis (Fig. 4A and B), focusing on accurately mapping the lesion and staging it before deciding on choosing a particular surgical procedure.

### Pediatric patients

We operated on 17 pediatric patients, aged 12 days to 17 years, mean 8.2 years, median 9 years (Table 2). Eight of them (47%) had associated co-morbidities that could potentially affect the postoperative course and eventually the outcome (except the hearing loss).

Subglottic stenosis was the most common encountered pathology followed by PGS and AGS. Therefore, the glottic/subglottic location of the stenosis was landmark of encountered advanced pediatric airway stenosis (71%), as such, most pediatric patients underwent LTR with cartilage rib graft (16/19 procedures). Most of these LTR's were performed as double-stage (11/16), mainly when anterior and posterior grafts were used simultaneously, when there was a need for relatively prolonged stenting, when a tracheostomy tube was needed to be kept in the postoperative period for controlling airway secretions in high risk patients (e.g. cerebral palsy, seizures, Down syndrome).

**Figure 5:**
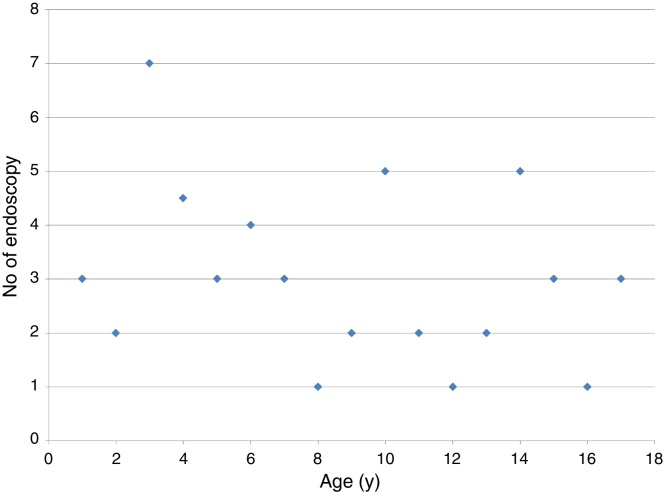
Number of follow-up endoscopies needed according to the age of the patient.

Stenting was frequently needed in the pediatric patients (16/19 procedures), with silicone-made stents being the most commonly used. This resulted in a high incidence of granulation tissue formation that was dealt with appropriately (Table 5). Tracheomalacia was encountered postoperatively only in pediatric patients, but was mild and did not require further intervention. Postoperative endoscopy was a crucial part of the care, ensuring close follow-up of the reconstructed area, removing any growing granulation tissue to prevent restenosis or formation of adhesions. Despite variability among patients, there might be a trend to need more endoscopies in the youngest patients (Fig. 5). All patients were decannulated except one with cerebral palsy, who needed the tracheostomy tube to stay in place to provide airway toileting (Table 2).

The voice was adequate in most pediatric patients. However, severe dysphonia was encountered in a one year old infant with Cohen IV AGS (patient 4, Table 2), where the vocal cords were found to be ill defined during the first surgery (Fig. 1B). She is currently receiving voice therapy and awaiting a trial of injection of hyalo-urinic acid to give bulk to her vocal folds and create adequate contact during phonation. A 17 year old girl also suffered from moderate dysphonia (patient 15, Table 2). She had a previous unsuccessful CO_2_ laser trial of excision of the web prior to referral to our clinic. Her vocal cords looked traumatized by that initial attempt and resulted in a persistent dysphonia. She is currently undergoing voice therapy.

### Adult patients

Eight adult patients were reviewed, aged 18–45 years, mean 25 years, median 22.5 years (Table 3). Only two patients had co-morbidities that did not affect the outcome, except for the quality of voice. In contrast to the pediatric patients, adult patients had more lesions affecting multiple levels, including supraglottic and tracheal. Multiple procedures were needed in 3 patients, including one patient that failed LTR twice and required a salvage CTR. The latter became the procedure of choice for adults with advanced LTS after encountering difficulties (e.g. infection, re-stenosis, delay in healing) using expansion procedures using rib cartilage grafts. Stents were used as frequently as in the pediatric patients for a comparable duration of time too, with surprisingly less granulation tissue formation. However, adult patients had other complications detailed in Table 5.

The postoperative voice of the adult patients was good in general. One patient (patient 7, Table 3) had gastroesophageal reflux causing intermittent mild dysphonia; it was treated by PPI with good improvement. Another patient (patient 2, Table 3) had moderate dysphonia secondary to pre-existing bilateral vocal cord paralysis (2ry to his initial neck trauma). He is receiving voice therapy to improve his phonation.

## Discussion

Congenital airway stenosis includes laryngeal atresia, laryngeal web, posterior glottic stenosis, subglottic stenosis and tracheal stenosis (complete tracheal rings). Most of these pathologies are believed to result from failure of recanalization of the airway during embryological development. Congenital subglottic stenosis is defined as a subglottic diameter of less than 4.5 mm in a newborn or less than 4 mm in a premature infant, in the absence of acquired causes of stenosis.^10^ It is the most common cause of congenital airway narrowing and the third most common cause of congenital stridor after laryngomalacia and vocal cord paralysis. It can be due to a cartilaginous malformation, a fibrous narrowing or a glandular hyperplasia. It tends to be milder than an acquired stenosis, having a better prognosis and allowing in some cases a wait-and-see policy.^3^

Acquired LTS is more common and results from prolonged endotracheal intubation in 90% of the cases. It is estimated that 1–5% of intubated children may eventually develop LTS.^11^ Other factors may include external trauma, inflammatory conditions or tumors. In children, the most susceptible area is the subglottis, as it is the narrowest part of the larynx, has a delicate mucosa and submucosa, and is formed of a complete cartilaginous ring.^3^ The posterior glottic/subglottic area can be another site of pathology as it may be subjected to direct pressure trauma from the endotracheal tube. Other sites of trauma include the trachea, due to balloon or tracheostomy tube injury, and the glottis secondary to intubation or external trauma.

In adults, LTS is usually acquired and is the result of intubation's trauma in more than 50% of the cases. Autoimmune disease and idiopathic etiology can account for 18% of the cases, each. The site of stenosis differs according to the etiological factor. The trachea, for example, is commonly involved in autoimmune and iatrogenic causes, while less affected in idiopathic etiology.^12^, ^13^

Treatment may include balloon dilatation, which has recently gained popularity and has been tried even in patients with advanced stenosis or as a primary treatment modality.^14^, ^15^ It was used in some of our patients but was not successful, resulting in the need of an open reconstructive procedure. Nonetheless, the use of balloon dilatation postoperatively might be beneficial to treat an early re-stenosis or stricture and prevent its progression into a more severe form.

In a systematic review of dilatation as a primary treatment modality for LTS, Chueng and Chadha (2013) reported a 50% success rate with balloon dilatation, which increased to 50–78% upon coupling it with adjuvant therapy.^16^ Recently, Günaydın et al. (2014) compared balloon dilatation to LTR as a primary treatment modality and noticed that balloon dilatation needs more repetitive interventions than LTR with a higher re-stenosis rate (63.2% vs. 31.3%).^17^ More concerns were raised in another recent comparative study by Maresh et al. (2014) who stated that there is a poor definition of the safety profile for balloon dilatation.^18^ They believe that the procedure carries risks of worsening the stenosis, affects airway tissue integrity, and in particular increases the chance of needing urgent airway intervention. Balloon dilatation has definitely its role but does not replace the effective role of LTR in providing a long lasting safe airway, especially in advanced stenosis.

We have shown in our study that LTR is an important tool in the airway surgeon's hand to repair moderate to severe laryngotracheal stenosis. Laryngotracheal airway surgery includes a variety of techniques depending on the site and extent of the airway pathology. The aim is to increase the airway lumen diameter and allow the patient to be decannulated as early as possible. Attempts to relieve such an obstruction started way back in 1956, when Rethi described posterior splitting or cricoidotomy with long-term stenting.^2^, ^19^ Anterior cricoid split was then performed by Cotton and Seid in 1980, to enable extubation of infants suffering from SGS. These procedures were later modified by introducing costal cartilage grafts with or without stenting the expanded area.^20^, ^21^ Since then, various corrective and reconstructive techniques have been described including cricotracheal resection.

Because laryngotracheal airway pathologies can affect different areas of the larynx and trachea, it would be important to use appropriate mapping of the lesion prior to deciding on the best reconstructive/corrective procedure. We have used a combination of assessment techniques to correctly map the location and extent of the lesions and this combined method proved to be valid and beneficial, especially that intraoperative finding corresponded to our preoperative topographic delineation of the lesion in all cases.

The various available staging systems are quite useful to correctly document the present stenosis. One should avoid using a single staging system to describe any type of stenosis, as this may lead to inaccurate description of the lesion and to inappropriate reporting of results.

The Cotton–Myer staging system^5^ is one of the most commonly used grading systems to classify an airway stenosis. Though it was devised to stage isolated SGS, it has been used in several reports to stage other stenotic areas, like tracheal and laryngotracheal, something we do not agree on or advise. A symptomatic isolated SGS often needs surgical intervention. It can be caused by a narrowing anterior shelf, bilateral lateral shelves or a circumferential narrowing. The mode of expansion will depend on the type of narrowing. An anterior shelf can be adequately corrected by an anterior cricoid split and a modified boat cartilage graft to maintain the expansion. It is often a single stage procedure that needs a short-term or no stenting, which is usually done using an endotracheal tube. A subglottis with circumferential stenosis or bilateral shelves are managed by an anterior and posterior cricoid split which are supported by a boat shaped posterior and modified boat shaped anterior grafts. The reconstructed area almost always needs stenting to stabilize the area while healing occurs. The duration of stenting will depend on the stability of the reconstructed area at the end of the procedure.

Stents are often a source of granulation tissue formation and care should be taken to monitor such a reaction to prevent restenosis or formation of obstructive adhesions. In our series, not every patient of the 21 who had a stent, got granulation tissue, and granulation tissue even occurred in some patients who had no stent (Table 2, Table 3). The ET tube was used in 10 patients and was not associated with granulation tissue formation, in contrast to the silicone stent that showed a reaction in 8 out of 12 patients in whom it was used. The age range was similar between both groups, but the mean duration of stenting was different (5 days for ET tube vs. 21 days for the silicone stent), reflecting the necessity to limit the stenting period. Looking specifically at the age of the patients, pediatric patients seemed to be more vulnerable to form granulation tissue than adult patients and thus should be more closely monitored with frequent endoscopies until resolution of the granulation tissue formation (Table 4).

Performing a single-stage or a double-stage operation relies on the ability to avoid a tracheostomy at the end of the procedure while achieving a safe airway. It also relies on the severity of the present pathology, and the stability of the airway. Including grafts during reconstruction would decrease the required stenting duration. Cartilage grafts are most commonly harvested from the ribs but alternatives include auricular, thyroid alar and septal cartilage.^2^, ^22^ Rib grafts are harvested with an intact perichondrium on one side to facilitate mucosalization. Their success in reconstructing the subglottic area exceeds that in correcting tracheal stenosis. They are also noticed to better integrate with the airway framework in the pediatrics than in adults. Adult's cartilage has foci of ossification which makes its carving more difficult, suturing it into the airway framework harder, and healing slower, with a possibility of acquiring an infection and extruding.

When the subglottic area is totally occluded (grade 4), the area cannot be expanded and is rather resected, hence the CTR. In addition, severe grade 3 stenosis, especially when framework fibrosis exists, is best treated with CTR.^23^ It is a more challenging procedure, but with a higher success rate.^24^ Though only few cases of CTR were reviewed in the current study, we have found this procedure particularly rewarding in the adult patients, where using of cartilage rib grafts is avoided.

When the stenosis involves both the larynx and the trachea, the management may include tracheal resection and anastomosis and or airway expansion using cartilage graft or CTR. In these cases, the reconstruction method is tailored specifically to the present pathology, according to the obtained preoperative topography of the lesion. These may be tough cases and decannulation may not happen following one reconstructive procedure (Table 3).

Looking at those cases that failed an initial reconstructive procedure despite adequate preoperative mapping, we could realize the following:

Two patients (patients 1 and 5, Table 3) were subjected to initial resection and anastomosis of the involved upper trachea, which resulted in aggravation of the existing SGS at the site of anastomosis (crico-tracheal junction). This necessitated additional reconstruction of the subglottic area with an anterior and posterior graft. These 2 procedures could have been avoided by performing a CTR from the start.

Patients 5 (Table 2) and 1 (Table 3) had an additional procedure (LTR with anterior graft) to correct a suprastomal collapse, which is often associated with a long standing tracheostomy tube.

Patient 8 (Table 3) taught us to avoid using rib cartilage grafts in subsequent repair of an adult airway stenosis. A CTR from the start would have spared the patient two additional major procedures.

Patient 4 (Table 2) was a challenging case and two procedures could not be avoided. The pathology was of what could be classified as partial laryngeal atresia. These are delicate and tough cases that are expected to require more than one procedure to reach a safe airway.

Isolated tracheal pathology are hard to stage as there is no single commonly used grading system that can assess all tracheal pathologies. When expansion is needed, we have found the auricular cartilage graft of great use both in pediatric and adults patients due to its appropriate contour and elasticity that conforms with the normal shape of the tracheal rings.^25^

Glottic stenosis is less common but can usually be managed successfully with a single surgical procedure. Again, using appropriate classification for each type of stenosis (anterior vs. posterior glottis) will ensure proper dissemination of information about the existing pathology among treating surgeons and in published reports.

Our overall decannulation or extubation rate was 96% which is comparable to that present in the literature, for both pediatric and adult patients.^2^, ^4^, ^9^, ^19^, ^23^, ^24^, ^26^, ^27^, ^28^, ^29^, ^30^, ^31^, ^32^, ^33^, ^34^, ^35^, ^36^ Operation-specific decannulation rate is a more common method of reporting success rate; however, it is a simple way that may overlook the type, location and extent of the lesion. The rates may get over-rated by including lesions that are of low stages (Rizzi et al.,^2^ Agrawal et al.,^19^ White et al.^33^). Performing a single stage or a double stage operation will depend on several factors already discussed above, and should not be a criteria for reporting success rate as done by some authors (Saunders et al.,^34^ Gustafson et al.,^35^ Rhee & Toohill^36^). We prefer reporting the success rate according to the type/site of pathology (like Rutter & Cotton^27^ and Wyatt & Hartley^28^) with emphasis on differentiating between treating mild (stage 1 or 2) vs. moderate to severe (stage 3 or 4) lesions.

Though both pediatric and adult patients had favorable outcome, it is worth mentioning that pediatric patients (especially the infants and young children) need more meticulous techniques during airway reconstruction due to the smaller dimensions of the airway and the tendency to form granulation tissue when a stent is used. Postoperative care in the intensive care unit adds another aspect to the challenges encountered in the pediatric patients regarding the need for sedation and tracheostomy/endotracheal tube care, and other medical treatment especially if co-morbidities exist.

To be transparent, it is very important to specify how many reconstructive/corrective procedures were needed to achieve decannulation. Requiring multiple procedures may reflect either the complexity of the case (e.g. multi-levels stenosis, co-morbidities) or the inefficacy of the used technique for the particular lesion. Details will be able to pinpoint the reason behind a particular failure.

## Conclusion

The review of our approach to open airway repair/reconstruction showed its efficacy in advanced-stage laryngotracheal stenosis. Good knowledge of a variety of reconstructive techniques is crucial to achieve good results in a variety of age groups.

## Conflicts of interest

The authors declare no conflicts of interest.
